# Ethnomedical survey of Berta ethnic group Assosa Zone, Benishangul-Gumuz regional state, mid-west Ethiopia

**DOI:** 10.1186/1746-4269-5-14

**Published:** 2009-05-01

**Authors:** Teferi Flatie, Teferi Gedif, Kaleab Asres, Tsige Gebre-Mariam

**Affiliations:** 1 Department of Pharmaceutics, School of Pharmacy, Addis Ababa University, PO Box 1176, Addis Ababa, Ethiopia; 2 Department of Pharmacognosy, School of Pharmacy, Addis Ababa University, PO Box 1176, Addis Ababa, Ethiopia

## Abstract

Traditional medicine (TM) has been a major source of health care in Ethiopia as in most developing countries around the world. This survey examined the extent and factors determining the use of TM and medicinal plants by Berta community. One thousand and two hundred households (HHs) and fourteen traditional healers were interviewed using semi-structured questionnaires and six focused group discussions (FGDs) were conducted. The prevalence of the use of TM in the two weeks recall period was 4.6%. The HH economic status was found to have a significant effect while the educational level and age of the patients have no effect either on the care seeking behavior or choice of care. Taking no action about a given health problem and using TM are common in females with low-income HHs. Forty plant species belonging to 23 families were reported, each with local names, methods of preparation and parts used. This study indicates that although the proportion of the population that uses TM may be small it is still an important component of the public health care in the study community as complementary and alternative medicine.

## Background

Since time immemorial, human beings have found remedies within their habitat, and have adopted different therapeutic strategies depending upon the climatic, phytogeographic and faunal characteristics, as well as upon the peculiar cultural and socio-structural typologies[1].

Ethiopian traditional medicine (TM) comprises of the use of plants, animals and mineral products as well as beliefs in magic and superstition, although ethnobotany is the major one[2,3]. Studies reported that a significant proportion of the Ethiopian population still depends on TM for its health care services[4,5] and more than 95% of traditional medical preparations are of plant origin[6]. Documenting traditional medical knowledge is important to facilitate discovery of new sources of drugs and promote sustainable use of natural resources. On the other hand, the knowledge of the factors involved in the selection of treatment options at household (HH) level is important for health service planning and to incorporating herbal medicine in a country's health care delivery system.

Despite its significant contributions, TM in Ethiopia has attracted very little attention in modern medical research and development, and less effort has been made to upgrade the role of TM practice[7]. This study, therefore, attempts to identify and document factors determining the use of TM and medicinal plants used by Berta ethnic groups, Assosa Zone, mid-west Ethiopia.

## Methods

### Study area

Benishangul Gumuz Regional State (BGRS) is one of the nine Federal States of Ethiopia located in the mid-western part of the country and having a total area of about 50,382 Km^2^. According to the 2001 Population and Housing Census of Ethiopia, the total population of Benishangul-Gumuz region was 460,459 which gives a population density of 9/Km^2^. Assosa zone, one of the three zones and two special Woredas (second from lowest administrative units in government structure) in the region, has a total area of 1,519 Km^2 ^and a population of 28, 970 (population density of 19.1/Km^2^).

**Figure 1:**
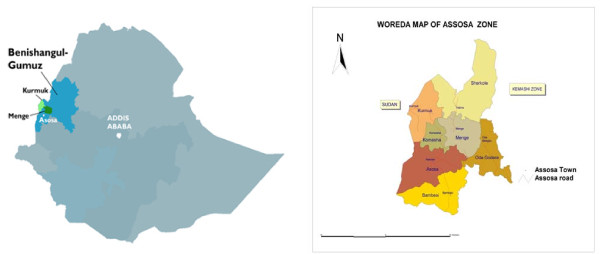
**Map of Assosa Zone (Finance and Economic Bureau, Benishangul-Gumuz Region)**.

The indigenous population of BGRS consists of five ethnic groups: Gumuz, Berta, Shinasha, Mao and Komo accounting for 23.4%, 26.7%, 7.0%, 0.6% and 0.2% of the total population, respectively. The Berta ethnic group resides mainly in the 7 Woredas of Assosa Zone (Figure 1) and more than 96.3% of the population of this ethnic group is Muslims[8].

The livelihood of nearly 95% of the population is subsistence farming. The enhancement of even this subsistence farming is precluded by the small number of livestock, which is commonly attacked by enzootic diseases, and frequently by paroxysms of epizootic episodes[9].

At the time of this survey, the region had 2 hospitals, 7 health centers, 75 health stations and 44 health posts of which 1 hospital, 3 health centers and 44 health posts are located in Assosa Zone[10,11]. In 2001, the top ten causes of morbidity in the region were; malaria (43.8%), helminths (13.6%), respiratory diseases (9.4%), dysentery (6.9%), gastritis and duodenitis (5.5%), rheumatism (5.3%), pyrexia of unknown origin (4.4%), skin diseases (4.1%), unspecified anemia (3.8%) and diseases of the digestive system (3.2%)[11].

### Data collection and analysis

The Institutional Ethical Board Review of the School of Pharmacy has given permission to conduct the study prior to the commencement of the survey. Information on demographic characteristics, prevalence of perceived illnesses, factors associated with preference of health care seeking options, medicinal plants used and hoarded as well as some healers' socio-economic characteristics were collected using two sets of semi-structured questionnaires – one for HH heads and the other for traditional healers. Moreover, focused group discussions (FGDs) were conducted with six heterogeneous groups with respect to sex, age and income levels. Each FGD consists of 7–9 members.

From the 7 Woredas of Assosa Zone, two Woredas namely, Menge and Komehsa were selected by simple random sampling technique. Proportionate to the size of the population, 7 Kebeles (lowest administrative units) from Menge and 3 Kebeles from Komehsa were selected randomly. The number of HHs included from each selected Kebele was again determined based on the size and identified using systematic random sampling techniques where every nth HH was taken until the required size was met in each Kebele. A total of 1,200 HHs were selected. Similarly, fourteen key informant healers were selected on the basis of their healing reputation with the help of Kebele administrators, health professionals in the area and community elders. Data collectors, who are high school students with knowledge of local language, were given training for two days on the data collection instrument.

Oral consent was obtained from each study participant before conducting the interview. Variables like socio-demographic characteristics of HH respondents, HH size, existence of illness during the past two weeks preceding interview date, choice of treatment options, names and parts of plants used, etc were entered in EPI info statistical software and analyzed.

## Results

### Summary of FGDs

Results of the six FGDs, conducted in six Kebeles of the two Woredas; Abora, Kudiyu and Belmeguha from Menge and Algela, Dareselam and Tselenkor Kebeles of Komesha Woreda are summarized below (the local names of illnesses written in italics and their major signs and symptoms or their closer meanings are shown in Appendix 1).

According to the respondents of the three groups, Menge Woreda, the major health problems identified by FGD participants were *Birde, Kulalite*, Malaria, *Gunfan, Ikek, Azurite, Kurtemat*, diarrhoea, *Cheguara *and *Ashmem*. In the other three discussions held in Komesha Woreda; malaria, *Gunfan*, *Ikek*, diarrhoea and *Birde *are listed as most prevalent illnesses in the localities. The common illnesses mentioned in both Woredas are similar, the only difference being the rare occurrence of some of these illnesses in Menge Woreda.

Most of the respondents of Menge agreed that modern medicine is the first choice during an episode of illness. They underlined that treatment recommended by modern medicine is strictly followed for the duration of the treatment and in case no improvement was observed from this treatment they would then resort to consulting traditional healers. Two respondents deviated from the opinions of members of their group in that for most of the illness episodes home made remedies were tried before going to health institutions and they even reported that for certain illnesses such as *Ashmem*, modern medicines are not believed to work at all.

Most of the respondents in all of the groups in Komehsa agreed that the choice of resorting to traditional healers depends on the specific illness episode to be addressed. For instance, modern treatment is the first choice for illnesses known to have been effectively cured by modern medicine. Malaria and pregnancy related problems are cited as best examples of illnesses with established signs and symptoms and which can be cured by modern medicine. On the other hand, *Setan beshita*, is believed to be an illness that can only be treated by traditional healers. Three respondents from two independent groups argued that for every illness episode, the use of home remedies is the first choice before consulting traditional healers. The patient only resorts to traditional healers only if the symptoms persist for two days or more following the administration of the home remedies. These deviants, however, agreed that modern medicine might be the first choice in case no one in the family or neighborhood claims to know TM for that specific illness.

Most of the medicinal plants, according to the respondents of Menge, are obtained from wild sources and there is no special protection or care given to these plants and they are treated just like any other plant that has no claims to medicinal values. Almost all of the respondents of Komesha also agreed that medicinal plants are obtained from wild sources except for some of the medicinal plants that are cultivated for added values. According to the respondents, the wild medicinal plants are treated just like other wild trees although some of these are even available only during the rainy season.

The members of all groups in Menge reported that the transfer of knowledge from generation to generation was by word of mouth, a practice that is being less commonly used these days. They admitted, however, that their forefathers collected and used medicinal plants for most of the illnesses that are treated today by modern medicine. The younger generation today has no or little interest in acquiring knowledge in TM and resort to it only after exhausting all treatment options by modern medicine.

All of the respondents in Komehsa agreed that knowledge of TM is given to mankind by God and transferred from generation to generation by word of mouth. However, they admit that this method for the transfer of knowledge is declining at a very fast rate from generation to generation. The respondents underlined that medicinal plants in use today are known to only a limited number of people in the communities and that the majority of users depend on either proxy knowledge or purchase herbs for illnesses they very well know to be effectively cured by herbs. This decline in the preservation of knowledge about TM is the result of lack of trust and confidence in the use of TM by the young generation on the one hand and, unwillingness on the part of traditional practitioners to share their traditional knowledge with the younger generation on the other.

The FGD participants reported that TM is an important alternative health care to the society for two reasons:

a) Some diseases are not treatable by modern medicines (for instance, *Ashmem*) for which TM is the only option and,

b) In some health facilities there is shortage of adequate diagnostic facilities and drugs, as a result of which appropriate treatment cannot be provided to the patients, who would then resort to TM.

As explained above, while the role of TM for the health care of the society is quite evident, traditional healers and members of the society believe that TM practice is not encouraged by the government which is in the opinion of the respondents tantamount to a criminal act.

### Perceived illnesses and patterns of resort

Among the 7,130 people in the HHs studied, a total of 570 illness episodes were reported, which gives a prevalence rate of 8.0%, in the two weeks recall period preceding the interview date. Females (57.5%), had more morbidity than males (42.5%). In response to the perceived illnesses, 85.8% went to health institutions, 3.5% went to healers and 1.1% used home made remedies while 9.6% took no action (Table 1). TM was found to be a more frequent choice of care for females (5.2%) than males (3.7%) with perceived illnesses in the two weeks recall period.

**Table 1:** Actions taken against perceived illnesses in two weeks recall period among members of Berta ethnic group Assosa Zone, June-July 2006

Demographic characteristics N (%)	No Action taken	Went to healers	Used home made remedy	Went to health institutions	Total
Sex					
Female	39(11.9)	13(4.0)	4(1.2)	272(82.9)	328(57.5)
Male	16(6.6)	7(2.9)	2(0.8)	217(89.7)	242(42.5)
Age					
≤ 5	11(5.9)	5(2.7)	0(0.0)	169(91.4)	185(32.5)
5–15	21(12.4)	3(1.8)	1(0.6)	144(85.2)	169(29.7)
15–65	22(10.8)	9(4.4)	5(2.5)	167(82.3)	203(35.6)
>65	1(7.7)	3(23.1)	0(0.0)	9(69.2)	13(0.2)

Total	55(9.6)	20(3.5)	6(1.1)	489(85.8)	570(100.0)

Among the respondents who claimed to have used TM, most (54.8%) believed that TM is more effective, 24.2% claimed the use of TM only when modern medicine failed, while 19.1% preferred TM because of its low cost and the remaining 1.9% claimed that lack of access to modern medicine prompted them to resort to TM.

### Factors associated with patterns of actions taken

As shown in Table 2, economic status of the HHs appears to influence the health seeking behavior and preference of treatment options. In this regard, there was a decrease in the percentages of no action respondents from 58.2% among low-income group to 30.9% and 10.9% among middle income and higher income groups, respectively. The economic status of the HHs was found to have a significant influence on whether to take actions or not during episodes of illnesses (X^2 ^= 9.98, P < 0.05) (Table 3).

**Table 2:** Effect of economic status on the action taken against illness, Berta ethnic members Assosa Zone, June-July 2006

	Economic status			
Action taken N (%)	Low	Middle	High	Total
No action taken	32 (58.2)	17 (30.9)	6 (10.9)	55 (100.0)
Went to healers	17 (85.0)	2 (10.0)	1 (5.0)	20 (100.0)
Used home made remedies	3 (50.0)	1 (16.7)	2 (33.3)	6 (100.0)
Went to health institutions	239 (48.9)	188 (38.4)	62 (12.7)	489 (100.0)

Total	291 (51.1)	208 (36.5)	71 (12.5)	570 (100.0)

**Table 3:** Factors affecting patterns of resort among Berta ethnic members, Assosa Zone June-July 2006

Associated Factor	Chi-Square	Yates corrected	Degree of freedom	P-Value
Economic Status	9.9795		2	<0.0408
Educational status (non-stratified)		2.0019	1	<0.1571
Educational status (low income stratum)		0.3952	1	<0.5296
Educational status (middle and high income stratum)		1.2496	1	<0.2636
Age of the ill (non-stratified)		9.4660	2	<0.0088
Age of the ill (low income stratum)		0.5248	1	<0.4688
Age of the ill (middle and high income)		0.0584	1	<0.8090
Sex (non-stratified)		5.1409	2	<0.0765
Sex (low income stratum)		4.0720	1	<0.0436
Sex (middle and high income stratum)		0.7509	1	<0.3862

The influence of education on treatment preference was analyzed and it was found that although non-literates tended to use TM more than literates, the relationship was not statistically significant (Table 3 and table 4)

**Table 4:** Preference of care of household respondents by their level of literacy of Berta ethnic members Assosa Zone, June 2006

Preference			
Literacy	Traditional practitioners	Modern health institutions	Total
Respondents without formal education	51	663	714
Respondents with formal education	23	444	467

Total	74	1107	1181

In the study community there was a preferential care seeking behavior both by sex and age in which children below the age of 15 are given priority over adults and males over females. The stratified analysis (income as stratification variable) showed that actions taken against illness had significant association with sex in low-income respondents, and age did not show significant association with action taken against illness (Tables 3 and 5).

**Table 5:** Actions taken by household respondents by socio-demographic characteristics of those with reported illness in two weeks recall period, Berta ethnic members Assosa Zone, June-July 2006

Variables	No Action	Used traditional medicine	Went to health institutions	Total
Sex				
F	39	17	272	328
M	16	9	217	242
Age				
≤ 15	32	9	313	354
> 15	23	17	176	216

Total	55	26	489	570

### Use of medicinal plants

A total of 40 species of plants with claimed medicinal values were collected and botanically identified during the course of this study. HH respondents reported the use of 37 plant species (Table 6) while only 10 herbs were found to be utilized by healers (Table 7). Among these plants, 28 are fully identified by their scientific names while 12 are identified at the genus level only. The identified plants fall under twenty-three plant families with the largest number falling under Fabaceae followed by Euphorbiaceae and Asteraceae.

**Table 6:** Medicinal plants reported by household respondents of Berta ethnic group, Assosa Zone, Benishangul-Gumuz Regional State, June-July 2006

Scientific name	Family	Vernacular name	Collection No.	Indication(Citation)	Part used	Preparation
*Acacia *sp.	Fabaceae	Mezel	TF-145	* *Gunfan *(4), Headache	Fruit	Ground, boiled with water and drunk
*Achyranthus aspera *L.	Amaranthaceae	Dumugelo	TF-031	Fever	Leaf	Boiled with water and drunk
*Acmella caulirhiza *Del.	Asteraceae	Etsegne andewu	TF-171	* *Tirse Himem *(4)	Root	Ground and put in-between teeth
*Aristolochia bracteolata *Lam.	Aristolochiaceae	Abujenajil	TF-186	Diarrhoea (2), * *Lib Himem *(3)	Seed	Ground, dispersed in water and drunk; also applied on the body
*Breonadia salicina *(Vahl) Hepper & Wood	Rubiaceae	Digel	TF-203	* *Yehitsan beshita*	Stem	Ground, dispersed in water and drunk
				* *Kurtemat*	Seed	Put into fire and exposed to its smoke
		Tigl		* *Setan* (2)	Stem Root	Dried stem and root are put in fire and patient is exposed to the smoke
*Bridelia *sp.	Euphorbiaceae	Sheketful	TF-200	Headache (4), * *Gunfan*	Root	Ground and applied on the head and drunk with water for *Gunfan*
*Calotropis procera *(Ait.) Ait.f.	Asclepiadaceae	Aberdade	TF-188	Antidot for scorpion bite (2)	Latex	Fresh leaves are cut and the exuding latex applied to affected area
*Calpurnia aurea *(Ait.) Benth.	Fabaceae	Etsegne eru	TF-194	* *Yehitsan beshita *(9)	Root	Ground and drunk with water
*Carrisa spinarum *L.	Apocynaceae	Etsegne gundew	TF-042	* *Yewegeb medhanit*	Root	Ground, dispersed in water and drunk
*Cissampelos pareira *L.	Menispermaceae	Abujelajil	TF-190	Diarrhoea (6), Abdominal cramp (1), * *Lib Himem *(3)	Root	Ground and drunk with water
		Etseyanefasu/di		* *Lib Himem*	Leaf	Ground, dispersed in water and drunk
*Clematis *sp.	Ranunculaceae	Etsegn egne	TF-202	Headache	Root	Ground and drunk with water
		(Etseye Egne)		* *Yetut medhanit*	Seed	Ground, dispersed in water and drunk; also applied on the affected area
		Shekedful		Snake repellant (2), * *Gunfan*	Seed	Ground, dispersed in water and sprayed in areas around house; also smoked like cigarette for * *Gunfan*
*Clerodendrum myricoides *(Hochst.) R. Br. ex Vatke	Verbenaceae	Bishchereh		* *Cheguara*	Root	Ground, dispersed in water and drunk
		Etsegne shaleha	TF-184	* *Birde*(2)	Fruit	Ground, dispersed in water and drunk; also applied on the affected area
		Etseya shalew		* *Birde *(2)	Root	Boiled with water and drunk like soup
		Etseye hoho		* *Gunfan*	Root	Ground, dispersed in water and drunk
*Combretum *sp.	Combretaceae	Keye	TF-206	Diarrhoea, * *Lib Himem*	Bark	Eaten as it is or ground, dispersed in water and drunk
*Croton macrostachyus *Del.	Euphorbiaceae	Abnga	TF-067	Anti-dot for snake and corpion venom (3)	Bark	Ground into powder and applied to affected area
*Dovyalis *sp.	Flacourtiaceae	Etseya bishu	TF-207	* *Lib Himem *(2)	Root	Ground and drunk with water or eaten as it is
*Echinops *sp.	Asteraceae	Etsegne setan	TF-185	* *Setan *(3)	Root	Dried, put in fire and patient is exposed to the smoke
*Flacourtia indica *(Burm.f.) merr.	Flacourtiaceae	Agnaneshewe	TF-204	* *Cheguara *(1), Malaria (1)	Fruit	Eaten as it is
*Grewia mollis *Juss	Tiliaceae	Hurinotse	TF-010	* *Dem mefses lemakome *(2)	Stem	Cut into pieces and put on the bleeding part together with the leaf
*Grewia trichocarpa *Hochst ex A. Rich.	Tiliaceae	Horgnatse	TF-195	* *Dem mefses lemakom*	Bark	Tied on the part to cover the cut and stop bleeding
*Indigofera spicata *Forssk	Fabaceae	Atahuna	TF-192	Nausea	Seed	Ground to fine powder and drunk with water
		Etsegne Murkewu		Chronic patient	Root	Ground, dispersed in water and drunk
		Etsegne shumegn		* *Lib himem *(2)	Root	Ground, dispersed in water and drunk
*Lennea *sp.	Anacardiaceae	Kuwa	TF-080	Diarrhoea, Bone fracture (2)	Bark	Ground, mixed with hot water and drunk; or tied around broken area for *Siberat*
*Melia azedarach *L.	Meliaceae	Almim	TF-199	Malaria (8), Headache (3)	Leaf	Leaf boiled with water and drunk
*Ocimum canum *Sims.	Lamiaceae	Beshiw	TF-191	To get dirt out of eyes (3), Tracoma (1)	Seed	Powdered seeds are sprinkled into the eye
				* *Lib Himem*	Leaf	Ground leaf is drunk with water
*Phyllanthus limmuensis *Cufod.	Euphorbiaceae	Aselfudi	TF-196	* *Setan *(4), * *Wetet Beshita *(1)	Root	Ground and drunk with water
*Piliostigma thonningii *(Schumach.) Milne-Redh	Fabaceae	Magel Mukul	TF-036	* *Setan*	Root	Dried, put in fire and patient is exposed to the smoke
*Plectranthus *sp.	Lamiaceae	Etsegne retuba	TF-201	* *Birde*	Root	Ground, dispersed in water and drunk
*Pseudocedrela *kotschyi (Schweinf.) Harms	Meliaceae	Aduruba	TF-197	Diarrhorea	Bark	Eaten as it is
*Pterolobium stellatum *(Forssk) Brenan	Fabaceae	Qudu	TF-205	Diarrhorea (2), * *Cheguara*	Root	Root (after removing the cover) boiled together with *Acacia *sp. in water and drunk
*Ricinus communis *L.	Euphorbiaceae	Ashenshemuke	TF-045	* *Seberat*	Leaf	Immersed in warm water and used to massage the area
*Securidaca longepedunculata *Fresen.	Polygonaceae	Shekede	TF-187	Headache (3)	Root	Ground and applied to the head
*Senna *sp.	Fabaceae	Etsegne eyu	TF-89	Diarrhoea (14), Bone fracture (1)	Stem	Ground and drunk with water for diarrhea; powder tied on fractured bone
		Aterha Ayune		Diarrhoea (4)	Root Bark	Root is ground, dispersed in water and drunk; bark is eaten as it is
		Etseya shemegna		* *Setan *(4)	Root	Dried root is put on fire and patient is exposed to the smoke
		Etsegne shalew		Bone fracture (2)		Root is ground and drunk with water and also tied around the fractured bone
		Umusihir		Abdominal cramp	Seed	Powdered seed is dispersed in water and drunk
						Bark is eaten as it is; powdered root is dispersed in water and drunk
*Syzygium guineense *(Willd.) DC.	Myrtaceae	Abulmitse	TF-008	* *Yehitsan beshita*	Stem	Powdered, dispersed in water and drunk; also applied on the body
*Tamarindus indica *L.	Fabaceae	Mala	TF-193	Malaria (10), Diarrhoea, Appetizer (2)	Fruit	Chopped, dispersed in water and the suspension is drunk
*Vernonia *sp.	Asteraceae	Etsene ahuha	TF-175	Diarrhoea	Root	Ground, dispersed in water and drunk
		Heten		Malaria (2)	Leaf	Boiled with water and sugar is added before it is drunk
*Vigna *sp.	Fabaceae	Etsegne Alhanser	TF-198	* *Kanser *(2)	Root	Ground and drunk with water
*Ximnea *sp.	Olacaceae	Bibi	TF-207	* *Gunfan *(6), * *Kufigne *(1)	Leaf	Eaten with salt
*Ziziphus mauritiana *Lam.	Rhamnaceae	Amurusam	TF-189	Open wound	Leaf	Leaves are ground, dispersed in water and applied on the wound
				Malaria, Diarrhoea	Seed	Seed is ground, dispersed in water and drunk

(Illnesses with asterisks are in local terms and the major signs and symptoms or closer meanings are presented in Appendix 1).

**Table 7:** Plants reported by healers of Berta ethnic group, Assosa Zone Benishangul-Gumuz Region, June-July 2006

Scientific name	Family	Vernacular name(s)	Collection no.	Indication/s	Part used	Preparation
*Achyranthus aspera *L.	Amaranthaceae	Dalecha Debes	TF-031	* *Shererit*	Leaf	Rubbed between palms and applied to the affected area
*Acmella caulirhiza *Del.	Asteraceae	Gutecha	TF-171	* *Shererit*	Leaf	Ground, mixed with sesame oil, and also applied to the affected area
*Aristolochia bracteolata *Lam.	Aristolochiaceae	Abujelalen	TF-186	* *Setan *(2)	Root	Ground, dispersed in water and drunk, and applied to body
*Calpurnia aurea *(Ait.) Benth.	Fabaceae	Estegne eru	TF-194	* *Yehitsan beshita*	Root	Ground, dispersed in water then drunk, and also applied on body
*Clematis *sp.	Ranunculaceae	Shekedful Shekelful	TF-202	* *Ebdet*	Seed	Powdered and put in fire with gum Arabic & patient is exposed to the smoke
				Headache	Root	Ground and applied on the head
*Ocimum urticifolium *Roth.	Lamiaceae	Anchebu	TF-101	* *Mich*	Leaf	Rubbed between palms and the fluid is applied on the affected area
*Piliostigma thonningii *(Schumach.) Milne-Redh	Fabaceae	Mekel	TF-036	Bloody diarrhoea	Root	Ground, dispersed in water and drunk
*Sterospermum kunthianum *Cham.	Bignoniaceae	Estegne eyo	TF-111	Diarrhoea	Root	Ground, dispersed in water and drunk
*Syzygium guineense *(Willd.) DC.	Myrtaceae	Bul-meste	TF-008	* *Hod himem*	Seed	Ground and applied on the painful area
*Waltheria indica *L.	Sterculiaceae	Albe	TF-121	* *Ebdet*	Root	Ground, dispersed in water then drunk, and also applied on the head

(Illnesses with asterisks are in local terms and the major signs and symptoms or closer meanings are presented in Appendix 1).

According to the HH respondents, root was the most widely used plant part (46.4%) followed by seed (14.3%), leaf (12.2%), fruit (11.2%), bark (7.7%), and stem (3.6%) while in the remaining (4.6%) combination of one or more plant parts were used. Healers also reported use of roots in 63.3%, seeds in 17.1% and leaves in 14.6% of the plants.

The major proportion of plants was collected from wild sources (77.0%) while 13.2% was cultivated and the remaining 9.8% was from both sources. Medicinal plants are stored by few HHs (11.7%) and of these plants, 30.8% are kept for unspecified period (long), 34.0% for one year and the remaining between one day and one year.

Diarrhea is frequently reported to be the disease which responds best to TM followed by malaria, evil eye, *Lib himem/Lib dikam*, headache, *Gunfan *and *Yehistanat beshita*. However, doses were not well established for most of the claimed treatments (71.6%) i.e. quantities were unknown or approximate in 58.6% of the plants and duration of treatment is undetermined in 13.0% of the cases.

## Discussion

A number of surveys indicated that some illnesses are believed not to be cured by modern health care. For instance, demon possession and infertility are typical health problems for which people visit traditional healers in Kalabo District, Zambia[12]. Similarly, in this study society, *Ashmem, Setan *and *Ebdet *are believed to be cured only by TM. Therefore, TM remains important component of public health care in the study community.

Even if there are variabilities among study designs, recall periods and seasonal variations in disease frequency and associated choice of treatment options, most studies proved high rates of TM use[13,14]. Contrary to these findings, the prevalence of herbal drug use was found to be low (4.6%) in this study. This could be due to either under-reporting of use as a result of community's belief that traditional practice is unlawful act or high prevalence of illnesses is believed to be treatable by modern care in the study period.

Even though low prevalence of herbal drug use was reported in this study, the reasons for preferring herbal drugs were perceived to be due to efficacy of TM, and perhaps also due to economic and geographic inaccessibility of modern medicine. These reasons of preference and the fact that more females (7.2%) prefer visiting traditional healers than males (5.6%) are consistent with finding of other studies conducted in different communities in Ethiopia[5,14,15].

This study showed an increase in the rate of "no actions taken" against illness episodes with a decrease in economic status (negative relationship) and this association is found to be statistically significant (P < 0.05). Other studies also came up with a statistically significant association between socio-economic status and choice of health care provider, and health care is less likely to be sought when the individual or HH is poor[16,17].

This study, consistent with a study conducted in Zambia[12], showed that educational level has no significant effect on the choice of health care, while other studies reported the existence of a statistically significant association between educational level and choice of health care provider[5,17].

In this study, a significant association existed between female and no-action taking during illness in the low-income HHs (P < 0.05). A study conducted in Nepal also indicated that illness reporting, choosing an external care, choosing a specific health care provider, and spending money to treat the sick child are all associated with sex of the patient[18]. Moreover, being a woman is more highly associated with visiting traditional healers than modern health facilities[5,19]. Even though a priority in resource allocation for children (<15 years) in preference to adults was reported by participants of group discussion, the association of the age of the patient with health care seeking pattern was not statistically significant in all income groups (P > 0.05).

Similar to other studies carried out in northwestern Ethiopia among the people of Shinasha, Agew-awi and Amhara, the family Fabaceae was reported to have the largest number of plant species used for medicinal purposes among the Bertha ethnic group[20]. Awas et al. [21] also reported that the Fabaceae is the most widely used plant family among the Bertha and Gumez people.

In agreement with other ethnomedical studies conducted in different parts of Ethiopia, the present study has also documented the roots as the most commonly sought-after plant parts[22-24]. Moreover, the results of the present study are consistent with reports in previous studies done in south-western Ethiopia where a large proportion of medicinal plants are collected from wild sources[25-27]. It is well recognized by conservationists that medicinal plants primarily valued for their root parts and those which are intensively harvested for their bark often tend to be the most threatened by over-exploitation[28]. Thus, it is recommended that an urgent and concerted action be taken to conserve widely used medicinal plants in general and those plants for which the roots constitute the primarily valued part in particular before they are lost irretrievably.

## Conclusion

In conclusion, Assosa zone harbors high diversity of medicinal plants most of which are rare species and seasonal plants. Despite the reported low prevalence of TM use, herbal medicine remains important component of public health care in the study community as it is the only option for some illnesses and also the next alternative when modern medicine fails. Since roots are the most widely used plant parts and plants are mostly collected from wild sources, the risk of loss of biodiversity in the Zone is high. Doses are not established or are approximate for most treatments and most herbs are stored for unspecified period. Thus there is a risk of treatment failure due to loss of potency, if any, during storage with possible formation of poisonous products. The risk of loss of indigenous knowledge appears to be high in connection with lack of transfer of knowledge among family members and friends. Therefore, it is important that the government create awareness among community members about the significance of preserving traditional knowledge and conserving medicinal plants before they disappear, and thereby ensure the rights of people to use their traditional practices which are known for their proven safety and effectiveness.

## Competing interests

The authors declare that they have no competing interests.

## Authors' contributions

TF coordinated data collection; performed data entry and analysis; wrote the draft manuscript.

TG, KA and TGM initiated the idea; involved in the design of the study; developed data collection instruments and corrected the manuscript.

## Appendix 1: Glossary of meanings of local terms for illnesses

*Anget ebtete *or *Tibi*: Swelling of the lymph nodes of the neck area

*Ashmem*: Illness characterized by swelling of finger tips followed by painful sore and leading to loss of finger if untreated. This illness is believed to be caused by accidental contact with a certain worm.

*Atintseberat*: Fracture of bone of any part of the body from any cause

*Ayene himem*: Eye disorder characterized by severe irritation and redness

*Birde*: Coughing and chest pain with or without fever and believed to result from being predisposed to a draft of air.

*Buda*: Evil eye

*Kanser*: Swelling and fluid accumulation around the neck or breast in ladies

*Cheguara*: Burning sensation in the upper alimentary tract (possibly esophagus or stomach)

*Dem mefses lemakom*: To stop bleeding from cuts

*Ye dem manes *or *Azurit*: Illness characterized by dizziness and loss of balance usually while trying to stand-up from a seated position or long exposure to strong sun (similar to symptoms of anemia)

*Ebtet*: Swelling of body part especially of the abdomen, face and legs

*Ikek*: Skin disorder characterized by sever itching sensation (similar to scabies)

*Gubet beshita*: Disorder characterized by yellow coloring of eyes and urine (jaundice)

*Gunfan*: Common cold

*Hod himem*: Abdominal disorder with unknown cause and signs and symptoms that cannot be well stated by the patient

*Hod nefat*: Stomach distension

*Hullum beshita*: Acute illnesses of unknown etiology

*Kebad tekmat*: Diarrhoea that is of high frequency leading to severe loss of fluid

*Korekore*: Illness characterized by circular swollen spots on the head with mild itching sensation and dry and shading skin and hair around the affected area

*Kuakucha*: Skin disorder characterized by discoloring spots with mild and intermittent itching sensation (A kind of fungal infection)

*Kufigne*: Illness characterized by fever, headache, throat pain and skin rash (measles like symptoms)

*Kurtemat*: Joint pain (symptoms like arthralgia)

*Lib himem *or *Lib dikam*: Symptoms characterized by total body weakness and intermittent shock

*Majerat beshita*: Initial pain at the back of the neck area with subsequent difficulty of neck movement

*Megetatemiya himem*: Pain and swelling in joint areas of bones (symptoms are similar to symptoms of arthritis)

*Mich*: Febrile illness characterized by fever, headache, skin rash and muscle spasm

*Nifase*: Draft and associated muscle pain

*Setan beshita*: Illness believed to be caused by demon possession and characterized by intermittent convulsion (similar to symptoms of epilepsy)

*Shererit*: Skin disorder characterized by skin rash (symptoms of herpes)

*Shinte lemiakatelew *or *Chebt*: Illness characterized by symptoms of burning sensation and pain during urination (a kind of gonorrhea)

*Tirs himem*: Dental pain with or without swelling of face area (might be due to tooth decay)

*Trachoma*: Infectious eye problem characterized by irritant eyes, itching and mucous secretion

*Wegeb himem *or *Jerba himem*: Severe back pain especially at the lower end of the spinal chord

*Wugat*: Acute pain of the abdominal and thoracic area

*Yehitsane beshita *or *Wetete beshita*: Diarrhoea and vomiting in children on breast feeding, believed to result from some abnormality in the mother's milk.

*Yematwolde set endetewolde*: Fertility promoter in ladies with total infertility

*Yemitil beshita*: Symptoms of seizure (epilepsy)
